# Biological Adaptations Associated with Dehydration in Mosquitoes

**DOI:** 10.3390/insects10110375

**Published:** 2019-10-28

**Authors:** Christopher J. Holmes, Joshua B. Benoit

**Affiliations:** Department of Biological Sciences, University of Cincinnati, Cincinnati, OH 45221, USA; benoitja@ucmail.uc.edu

**Keywords:** Desiccation, phenotypic plasticity, local adaptation, mosquito-borne disease, vapor pressure, Diptera, Culicidae

## Abstract

Diseases that are transmitted by mosquitoes are a tremendous health and socioeconomic burden with hundreds of millions of people being impacted by mosquito-borne illnesses annually. Many factors have been implicated and extensively studied in disease transmission dynamics, but knowledge regarding how dehydration impacts mosquito physiology, behavior, and resulting mosquito-borne disease transmission remain underdeveloped. The lapse in understanding on how mosquitoes respond to dehydration stress likely obscures our ability to effectively study mosquito physiology, behavior, and vectorial capabilities. The goal of this review is to develop a profile of factors underlying mosquito biology that are altered by dehydration and the implications that are related to disease transmission.

## 1. Basics of Desiccation Resistance in Mosquitoes

**Figure 1:**
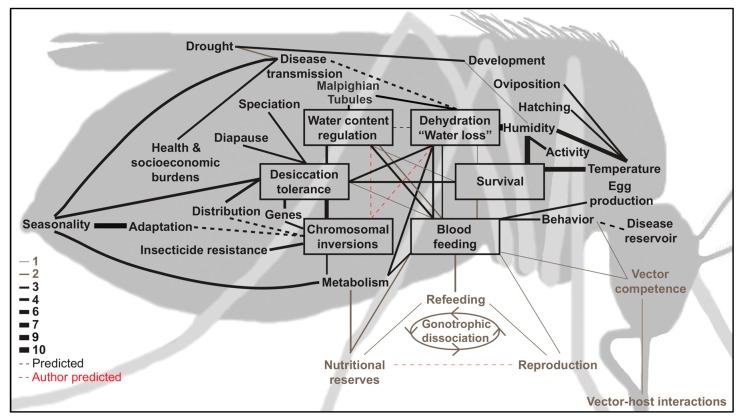
Concept mapping of factors integral to the dehydration of mosquitoes. Black line color signifies published support from three or more articles; grey, two or less articles; and, red, associations proposed in this review article. Solid lines denote at least one known association connecting two terms, dashed lines denote predicted associations from this publication (“Author predicted”) as well as predictions from other publications (“Predicted”). Line thickness is directly associated with the number of publications discussing connection of two terms. Associations are causal and supported by three or more publications, specific reference numbers are enumerated in Table S1. Connections with support from two or less publications are included to emphasize associations that are likely important to the holistic impact of dehydration on mosquito-borne disease transmission. A full account of associations is given in Table S2.

Pathogens that are transmitted by mosquitoes cause over 700 million human infections and more than one million deaths each year [1]. These diseases are complex and they carry tremendous health, social, and economic burdens, the impacts of which are inevitably underestimated [2,3,4,5]. Water loss has been directly implicated in altered mosquito behavior with predicted increases in West Nile virus (WNV) transmission [6], but it is also linked to reduced survival and oviposition [7], reduced nutritional reserves and egg production [8], and shifted geographic and microgeographic distributions [9] (Figure 1, Tables S1 and S2). Mosquitoes are highly susceptible to water loss as a result of high rates of evaporation through respiratory spiracles and the cuticle [10]. However, water loss can be countered by alterations in body size, metabolism, cuticular hydrocarbon composition, and by a number of behavioral adaptations [11,12,13]. These behavioral adaptations are likely present in all mosquitoes, but they may be intensified during adverse conditions, such as cold, heat, or drought. Over-wintering (diapausing) mosquitoes display an increased desiccation resistance that confers a reduced water loss rate [11,12,13], while a number of Anophelinae mosquitoes have adapted to survive hot and arid climates via long-distance migration or aestivation over the summer [14,15]. Interestingly, some *Anopheles* species are more susceptible to water loss, while other species have developed comparatively superior desiccation resistance [16,17]. Dehydration stress has resulted in genetic alterations and behavioral adaptations (both long- and short-term) that are integral to mosquito physiology, survival, and distribution [9]. Dao et al. postulated that the ability to exploit arid environments may have driven the divergence between many mosquito species, although species-specific adaptations are apparent [14].

### 1.1. Water Homeostasis Is Paramount for Mosquito Survival

Mosquitoes are under constant homeostatic strain, be that through unfavorable environmental conditions, immunological response to pathogens, or osmotic imbalances from blood feeding [9,18,19,20]. Mosquitoes navigate this suite of stressors with remarkable efficiency by seeking free water, nectar, hibernacula, and bloodmeals, and allow for their establishment as world’s deadliest animal [21,22]. Female mosquitoes that vector diseases are hematophagous and complete vitellogenesis following blood ingestion. However, mosquitoes encounter a plethora of osmotic pressures that are associated with blood feeding as a result of increased sodium content from blood plasma, increased potassium, and iron content from red blood cells, along with a massive influx of water [19]. Mosquitoes utilize a highly efficient renal excretory system to regulate osmolality and ion imbalances [10]. Following a bloodmeal, increased rates of excretion through diuresis are observed as a means of eliminating non-essential components of a bloodmeal. The appropriate regulation of water volume and ion concentration permits bloodmeal retention/utilization, decreased mortality, and an increased desiccation resistance between bloodmeals. Although desiccation tolerance is an integral part of mosquito survival, the biochemical and physiological mechanisms underlying tolerance (e.g., spiracular occlusion) remain understudied, even though significant progress has been made [23].

### 1.2. Current (Mis)Understandings of Mosquito Dehydration and Resulting Consequences in the Field

Underestimates in disease transmission rates likely stem from overlooking specific factors that are related to mosquito blood feeding behavior [24], modeling parameters [25,26], and host immunity [27]. An increased understanding of arthropod-borne disease dynamics might be specifically achieved through additional research in pathogen reservoirs [28], vector competence to various pathogens [29], barrier(s) to midgut infection [30,31], vector stress-tolerance mechanisms [32,33], climatic and environmental factors [33,34,35,36,37], microclimates [38,39], as well as factors that are involved in dry season and desiccation stressors [7,17,40]. Despite extensive studies on the relationship between temperature [25,39,41,42,43,44,45,46] and modeling of climate change [26,34,35,47,48,49,50,51,52,53,54,55,56,57,58,59] on the dynamics of arthropod borne diseases, few studies have investigated the impact of environmental stress in larvae [33,60,61] or the effects of humidity and drought [6,7,36] on eggs, larvae, or adults. Consequently, the specific drivers of arthropod-borne virus (arbovirus) seasonality [62], dry season ecology on mosquito behavior [40], and specific underlying mechanisms for survival in arid environments [63] have only been cursorily examined in a few species. Numerous environmental factors alter the distribution and propagation of arthropod-borne diseases, but three major environmental factors—temperature, precipitation, and relative humidity (RH)—are extensively used for disease outbreak predictions [54]. Despite modeling use, underlying mechanisms by which temperature, precipitation, and RH directly impact mosquito biology (e.g., dehydration stress, behavioral shifts) are not well understood [6]. These interactions are particularly relevant, as global aridity and drought are predicted to intensify and they become more unpredictable in the coming decades [64,65].

Mosquito hydration status, which can be influenced by temperature, precipitation, and RH, should be accounted for in the design of general mosquito studies and should be included in future disease transmission models. This review examines the importance of mosquito hydration status, how it may impact vector research outcomes, and offers recommendations for how this information might be applied. For example, two studies that utilized nearly identical experimental designs found entirely opposite results on the effect of trypsin inhibition on infection with Dengue virus (DENV). The discrepancy was attributed to the source of the trypsin inhibitors used [66], but another discrepancy between the studies was that of their blood feeding methodology. One study withheld sucrose (10% sucrose in H_2_O) and water for 36 h prior to blood feeding [67], while the other typically utilized methods that only withheld sucrose between one night and 24 h before blood feeding [68,69]. It remains unclear which of these factors underlie the discrepancy, but the dehydration of experimental animals should be avoided in future studies as a mechanism to promote feeding, since it is a stressor and likely alters subsequent blood feedings and bloodmeal retention. Although this is an extreme example, there are constant discrepancies in vector research relating to dehydration, both in lab- and field-based studies.

### 1.3. Behavioral Plasticity Is Relevant and Important in Response to Mosquito Dehydration Stress

Environmental factors prompt phenotypic alterations and plasticity where mosquitoes must maintain a balance between nutrition, hydration, reproduction, and survival. These factors are influenced by a complex interplay between environmental factors, which underlie molecular, physical, and biochemical regulation, as well as behavioral alterations (Figure 1, Tables S1 and S2). One of the most important factors for terrestrial organism survival is the ability to obtain sufficient water before succumbing to dehydration-induced mortality [70]. Water content regulation is an integral component of terrestrial evolution and it is of considerable interest, especially in regards to global climate change [71]. Unfortunately, the specific mechanisms by which mosquitoes regulate water content, survive, and continue to act as effective vectors are not thoroughly understood.

It is probable that dehydration prompts phenotypic alterations that mosquitoes rapidly counter with compensatory mechanisms [6]. The punctual response by mosquitoes to counteract water loss could offer one explanation as to how the effects of dehydration stress have remained relatively unnoticed and understudied. Although these stressors and compensatory mechanisms may appear to offset one another, it is likely that, through compensating for the lost water, the effects of dehydration and hydration mechanisms cause alterations in other facets of mosquito biology that contribute to disease transmission dynamics. For example, mosquitoes that were subjected to stress by DENV-2 infection showed a decreased motivation to blood feed, but exhibited an increased instance of refeeding [72]. Taken alone, these two factors appear as if they may result in a negligible net difference to vectorial capacity. However, compounding factors, such as DENV-2 infection and dehydration, which have been shown to increase blood feeding [6], may elicit interactions between stressors and prompt unique phenotypes. Compounded stressors may also affect survival and ultimately contribute to significant alterations in disease transmission dynamics. Although the net contributions of stressors to disease transmission rates may be minuscule or even null, it has been postulated that water content regulation and hydration-balancing mechanisms are associated with fitness tradeoffs and they could be exploited for vector control [9]. That is, if the mechanisms of action are more thoroughly understood.

### 1.4. A Plethora of Factors Influence Mosquito Biology and Interactions between Stressors Should Be Scrutinized

Although rehydration might occur through the acquisition of free water (not atmospheric sources, which ticks utilize [16]), distinct advantages are granted to mosquitoes that seek hibernacula to limit water loss [73]. Favorable microclimates (e.g., shrubs) can contribute to mosquito distribution, abundance, and survival [74], and they have also been shown to provide substantial breeding sites (e.g., treeholes) for Dengue vectors [75]. Concerning disease transmission, interactions between stressors, such as starvation and dehydration, and mitigating strategies that are employed by mosquitoes should be considered in control efforts [76,77]. Plants also need be considered for their source of nectar and as a source for rehydration [78,79,80]. Sugar acquisition is variable between mosquito species and it is influenced by the presence of plants, alternative sources of water (i.e. free water or blood), nutritional reserves, sex, aridity, control efforts, and starvation [78,79,80,81]. In some populations, sugar meals are taken often, in others sugar is rarely taken, and in populations with a high prevalence of imbibed fructose, recorded frequencies may be deceptive [78,80,82]. An interplay between hydration status, sugar feeding, and blood feeding exists, with an antagonistic interaction between sugar and blood feeding that influences survival, vectorial capacity, fecundity, longevity, and behavior [78,81,82]. When sugar is withheld but free water is accessible, mosquitoes survive longer than those experiencing starvation and dehydration in tandem [6,83]. Likely in an effort to curtail the stress, starved mosquitoes become more active [84], which has similarly been postulated as one reason for the increased activity in dehydrated mosquitoes [6]. These behaviors underscore the close similarities between these stressors [85] and caution is advised for analyses involving starvation and/or dehydration, especially when blood feeding behaviors are incorporated.

Species, such as *Ae. aegypti*, are highly anthropophilic and they may have been driven to domesticity by increasing aridity (low RH). Where, through cohabitation, they developed a preference for blood feeding on humans, which are more readily available in dry, urban environments than other free water sources, such as nectar or standing water [86]. By exploiting human hosts and their domiciles, *Ae. aegypti* may reduce metabolic rates up to 2.5-fold by resting in cool (25 °C) reprieves, and may increase survival by one to four additional days through taking a blood meal [87]. Relative humidity also impacts mosquito activity dynamics, while considering that daily differences in relative humidity are sufficient enough to alter *C. nigripalpus* activity in ecological settings [88], and may likewise alter the activity of mosquitoes in general [6]. While, in the shade at 25 °C, humidity can considerably fluctuate from 50–95% RH [89], which corroborates the claims that daily humidity fluctuations are substantial enough to alter mosquito physiology and behavior [6,89]. Similarly, dry season hourly temperatures in West Africa varied both indoors and outdoors from 16–40 °C and relative humidity varied substantially from 5–95% [15]. Driven by temperature and humidity, mosquito resting behaviors are confined to warm and dry sites during cold months; cool and damp sites in the hot, arid months; and, in cooler, humid periods, mosquitoes are often observed in the open, likely for increased exposure to sunlight [90]. Temperature and moisture are important determinants for mosquito survival [91,92,93] and numerous interactions between these factors are discussed in 4.1, but avenues for further research on indoor biome impact remain unexplored [94].

## 2. Adaptations to and Influences of Mosquito Dehydration Dynamics

### 2.1. Wet and Dry Season Dynamics

Rainfall and relative humidity have been extensively used in disease and dispersion models (discussed in 1.2 and 3.3), especially in regards to wet- and dry-seasonality. Far fewer studies have investigated the underlying mechanisms that drive the observed model relationships. The direct effects of drought on survival, blood feeding, and larval maturation that likely contribute to disease transmission include: (1) drawing hosts and mosquitoes into close proximity due to scarcity of water [49], (2) increasing larval abundance and survival in semi-permanent habitats by reducing predators and competitors [95], (3) improving larval development by concentrating nutrients in water pools [54,59], and (4) delaying drainage system flushing permitting the development of larvae to adulthood [54,59,96]. Rainfall impacts oviposition, abundance, and survival by: (1) creating water pools for mosquito oviposition and subsequent larval development [15,50], (2) dampening the ground to provide increased humidity near the surface and act as a source of water for drinking [50], (3) dilution of larval resources to decrease survival [97], (4) elimination or dilution of pyrethroids to increase survival and likely bolster insecticide resistance in subsequent seasons [98], and (5) increasing the relative humidity during the resting and host-seeking periods. Understandably, oviposition is principally modulated by water availability with considerable contributions from humidity and rainfall [97]. Incorporating all of these factors, including the broad influences of seasonality, is important in understanding the biology and ecology of mosquitoes.

### 2.2. Long-term Adaptations for Survival During Unfavorable Periods

#### 2.2.1. Diapause, Migration, Aestivation, and Likely Anthropophilia, Promote Survival and May Establish Disease Reservoirs During Adverse Seasons

Transitory periods between the dry and rainy seasons or during winter contribute to marked phenotypes and a number of behavioral adaptations that promote survival. In addition, these dormant individuals likely serve as reprieves for mosquito-borne diseases, specifically viruses [99]. Female diapausing *C. pipiens* are conferred an increased resistance to desiccation [11], and *C. salinarius* females are able to survive a few months while overwintering and they emerge in the spring with blood feeding rates over 90% [100]. Despite similarities in desiccation tolerance, the water maintenance strategies employed during diapause differ from those that are used in over-summering species, such as *An. gambiae* and *An. coluzzii* [101]. Within the *An. gambiae sensu lato* complex, over-summering is likely accomplished by *An. gambiae* through migration, by *An. coluzzii* via aestivation, and *An. arabiensis* may potentially migrate or aestivate [14,15]. Recently, rapid, long-distance movement has been described in *Anopheles* [102], which could allow the movement of individuals to more favorable areas in short periods (less than one night). During aestivation, *An. coluzzii* can temporarily move to supplement water stores from bloodmeals, sugar sources, and pooled water [103]. Thus, *An. coluzzii* likely amplifies infections during dry seasons between June and September [14]. Likewise, ecologically unfavorable challenges prompt adaptations, such as the breaking of diapause for a bloodmeal by *Culex salinarius* [100], and summer aestivation in lieu of migration by *An. coluzzii* [14]. This likely permits both of these species to act as a reservoir for arthropod-borne disease during unfavorable conditions. However, due to the strenuousness of aestivation and the resulting population bottleneck, the control efforts during this time may substantially reduce peak malaria transmission levels [14]. Similar control efforts may be applied to WNV vectors (*Culex spp.*) during overwintering diapause in temperate regions [100].

However, control efforts are not ubiquitous. Humidity and water availability are two of the most limiting factors in small terrestrial insects [104] and humidity is also one of the most variable factors, regardless of urbanization [105]. Urban settings further complicate control efforts, where desiccation resistance, abundance, and distribution are increased for *Aedes* in urban areas [106]. Desiccation resistance can differ down to the species-strain level [104] and adaptations may result from locality- as well as niche-specific influences. Ecological influences also contribute to the propensity of some species towards anthropophilia or endophilia, where limited distance between the mosquitoes and hosts is advantageous. Although no obvious trends in the physiological and molecular variations were observed in the highly anthropophilic species, *An. coluzzii*, of similar geographic origins, local adaptations persisted, and may have been influenced by specific microhabitats [107]. It is likely that the underlying adaptive mechanisms that are required to live in arid environments may have driven other highly anthropophilic species (e.g., *An. gambiae*) towards anthropophilia and/or endophilia.

#### 2.2.2. Examination of the Genetic Components in Desiccation Resistance Offers a Glimpse into the Mechanistic Underpinnings

Studies in *Drosophila melanogaster* may be useful for creating a conceptual framework due to the lack of substance regarding desiccation-driven genetic alterations in mosquitoes. After undergoing over 100 generations of desiccation selection, *D. melanogaster* females were no more tolerant of water loss (i.e. percent water lost at death), but displayed a two-fold increase in survival, expanded their bulk water content (30% higher), and water-loss rates that were 40% lower than that of the controls [108]. These shifts were attributed to reductions in cuticular permeability, respiratory and excretory water loss, longer cuticular hydrocarbons, and increased glycogen content [108]. In addition to the alterations that were observed in *D. melanogaster*, mosquitoes have a heightened adaptive potential for desiccation resistance due to water content regulation associated with blood feeding and subsequent excretion (i.e. diuretic) behaviors [10]. Even within a species, female mosquitoes may be more adaptive as a result of their blood feeding behaviors [81]. These inherent feeding-excretion dynamics likely permit additional mechanisms, such as increased water retention from a bloodmeal, to increase desiccation resistance between feedings. Desiccation resistance phenotypes in mosquitoes likely result from the accumulation of low humidity adaptations over time, which encourage adaptation to, and exploitation of, arid environments, where increased success is expected to increase human-mosquito interactions.

#### 2.2.3. Chromosomal Inversion Polymorphisms Underlie Climatic Adaptations

##### Climatic Adaptation and Distribution

Mosquitoes belong to the one of the most recently diverged insect orders, Diptera, which radiated from a common ancestor approximately 240–250 million years ago (mya) [109,110,111]. The split between the Culicidae family and other dipterans constitute one of the most ancient divergences of extant fly lineages [112]. The Culicidae family is comprised of the Culicinae and Anophelinae subfamilies, which separated between 150 and 200 mya [109,113]. Unfortunately, mosquito-borne diseases have coevolved with mosquitoes, exhibit highly efficacious infectivity, and may offer advantages to the mosquito [114,115], which makes control efforts exceptionally difficult [116]. Therefore, understanding these vectors, the environmental conditions they experience, and their associated pathogens, is of the upmost importance.

*An. gambiae* and *An. coluzzii* in arid regions have chromosomal inversions (2La and 2Rb) [9] that have been linked to thermal and desiccation resistance, body size [117], and are consistent with aridity tolerance mechanisms [18]. A majority of inversions are under environmental control and likely contribute to aridity and local adaptation, habitat range, desiccation tolerance, as well as various other roles [9,118,119,120,121,122]. These contributions also appear to permeate other mosquito vector systems and they may contribute to some of the variation in *Anopheles* competence for *Plasmodium* [121]. It is possible that other adaptations, such as those that are related to elevation in Indonesian Dengue vectors [123], may contribute to elevation adaptation in *An. gambiae* and *An. coluzzii* via similar chromosomal inversion polymorphisms (in 2Rb) [122]. Inversion polymorphisms among local populations may fluctuate temporally, contingent upon wet and dry season dynamics [124], which may explain the developmental acclimation to dry seasonality, increased survival, and variability in the molecular form types that were observed in *An. gambiae* [125]. Such alterations may be related to the chromosomal inversions underlying increased basal metabolic rates and limited energy distribution during sub-lethal thermal and desiccation stress in *An. gambiae* [18]. An increased mechanistic understanding is severely wanting when considering the known associations between mosquito tolerance mechanisms, distinct phenotypes, metabolism, and distribution with chromosomal inversions.

##### Gene Regulation and Metabolism Related to Chromosomal Inversions

Inversions in 2La are associated with energetic dynamics that underlie behavioral and physiological alterations [18], where alterations in energetics and behaviors were suggested as means for metabolic and energetic balancing. The balancing of which occurred via the differential expression of sirtuin genes, altered locomotion, and shifted blood feeding frequency associated with the expression of the circadian rhythm genes *takeout 1* and *3* [18]. Candidate genes that are associated with the 2Rb inversion include a ligand-gated transmembrane ion channel encoding a voltage-gated calcium channel, which may influence mosquito courtship or neurophysiology [118], as well as numerous genes that are associated with DNA-repair and ATP production [18].

It can be expected that inversion clines are to be maintained via fitness and fecundity tradeoffs, where direct climatic interactions can be found in 2Rb polymorphisms and 2La inversions [18]. Chromosomal inversions have facilitated the exploitation of stressful climatic conditions, which have indirectly contributed to the profound and lasting repercussions that are characterized by increased vectorial capacity in previously insupportable regions [18]. Overall, the holistic importance and impact of chromosomal inversions on mosquito biology has yet to be established, but the incorporation of all factors integral to mosquito biology (e.g., reproduction, blood feeding, survival, water content regulation, etc.) should meaningfully bolster our understanding.

### 2.3. Adaptations to Dehydration Confer Biological Advantages

#### 2.3.1. Spiracule and Cuticular Hydrocarbon Adaptations Impact Adult Desiccation Resistance

Cuticular hydrocarbon (CHC) abundance is one major factor in the desiccation tolerance of *An. coluzzii* [103] and it further supports the suggestion of dry season aestivation by this species [101]. CHC differences were less apparent in *An. gambiae*, which supports a migratory hypothesis for these mosquitoes [101]. Regardless, the 2La chromosomal inversion in *An. gambiae* and *An. coluzzii* drives the cuticle thickness and CHC composition that constitute the desiccation resistance phenotype [9]. It also appears that a higher body water content results in greater desiccation resistance and increased survival, since *An. arabiensis* and *An. gambiae* both died at the same minimum water content [126]. Phenotypic plasticity in desiccation-resistance traits is embodied in dry season-*Anopheles* dynamics and it is probable in other environment-mosquito systems, such as in *Culex spp.* during diapause.

Another characterized long-term adaptation to desiccating environmental conditions is the structural occlusion and shortening of the mesothoracic spiracules. Spiracular occlusion has been suggested to progressively develop, vary between members of the same species, and ultimately minimize the metabolic demands and water lost to respiratory processes [63]. *An. stephensi* have showed that the spiracular length is shortened in summer months when compared to the post-summer and monsoon seasons and may be used as a taxonomic tool for the identification of ecological variants [127]. Continued investigation of basic physiological and structural differences might increase the effectiveness of discriminating between ecological variants and might also help to identify populations with an increased susceptibility to control methods.

#### 2.3.2. Metabolic and Transcriptomic Alterations Caused by Dehydration Affect Processes Integral to Mosquito Biology

Recent studies have begun to investigate the underlying mechanisms that contribute to desiccation resistance and understanding the genes that are responsible for water content regulation is of extreme importance since mosquitoes are highly susceptible to water-loss [16]. Differential gene expression has been postulated as the mechanism by which insects attain the phenotypic plasticity necessary to survive desiccation [128]. The dehydration of *C. pipiens* resulted in increased blood feeding propensity that was underpinned by alterations in carbohydrates and glycogen [6]. The results were similar in a dry-season ecological study that found alterations in glycerol phosphate, glucose-6-phosphate, and fructose-6-phosphate were important, which was consistent with increased protein or glycogen breakdown or as an indicator of increased pentose phosphate pathway activity [107]. Corroboratively, within the *An. gambiae sensu lato* complex, the dry season metabolic profiles were markedly similar and showed suppression in metabolic and reproductive processes, which likely bolstered adaptive potential and survival through alterations in cuticular, metabolic, and behavioral traits [129].

Biochemical and physiological alterations could be theoretically used as markers for the onset of the dry season as they have been utilized to support the position that different adaptive techniques exist in *An. coluzzii*, where certain metabotype differences represent “strong” aestivation abilities [107]. Likely similar to those that were observed in dry seasons, bouts of desiccation increased the transcript expression for DNA repair, stress response, and reactive oxygen species (ROS) detoxification, as well as the genes that underlie flight, like flightin and myosin [128]. Despite the wealth of knowledge that is provided by whole-organism transcriptomics, metabolomics, or proteomics, connecting the results to physiological alterations and increasing specificity is paramount. Correspondingly, extensive comparative transcriptomics in *Ae. aegypti* that were infected with WNV, DENV, and yellow fever virus (YFV) revealed that gene expression profiles may be conserved between, or specific to, mosquitoes infected with varying pathogens, and they are informative as well as tissue-specific [130]. Mosquito dehydration and disease transmission dynamics are moderated through a number of catabolic and metabolic pathways; knowledge that may be used to identify overlapping effects and, consequently, harbors the potential for vector control. It is important that tissue-specific evaluations be conducted on mosquito tissues at the interface of dehydration and disease transmission, such as at the midgut or salivary glands.

#### 2.3.3. Nutritional Reserve Dynamics Underlie Many Behavioral Adaptations

Previous research has postulated the presence of behavioral adaptations that allow for the reallocation of nutrients from a bloodmeal and repeated blood feeding to restore nutritional reserves [131]. These trade-offs lead to compromises between fecundity and metabolic reserves in relation to mosquito size [83], where similar relationships have been prompted by water stress in dehydrated mosquitoes. A simple mechanistic model is that water stress resulting in reduced nutritional reserves will decrease egg production [8]. However, a bloodmeal might be utilized for nutritional supplement [132], provide enough nutrients for survival until oviposition [133], and might directly aid in the short-term survival of drier climates through rehydration potential [6]. Although bloodmeal ingestion as a rehydration mechanism is contested [6,36], the ingestion of blood prompts water regulation mechanisms, is integral to mosquito biology, is required for pathogen acquisition and transmission, and it does likely act as a source for rehydration.

Blood is composed of approximately 80–85% water in humans (e.g., vectors = *Ae. aegypti*, *An. stephensi*) and about 87% water in chickens (e.g., vector = *C. pipiens*), with ~90% of the remaining dry weight being composed of mostly protein [134,135]. Mosquitoes may excrete over 40% of a bloodmeal within the first 1–2 h after blood feeding due to osmotic and mobility constraint [19,136], while regulating the hydration and nutrient levels for survival and reproduction. Mosquitoes are hygric organisms, are highly susceptible to dehydrating conditions [16], and must retain ample amounts of water to resist dehydration [137]. Obviously, there are a number of stringent mechanisms for (re)hydration present across mosquito species, but they are not thoroughly understood. We postulate that dehydration stress drives the depletion of water and nutrients as well as the compensatory mechanisms that are required for offsetting the detriments. It has been suggested that, although mosquitoes rehydrate from free water or nectar, temporal water pools and nectar production are likely reduced during times of drought and rehydration via host feeding could be more efficient [6]. Although recent studies have begun to explore the mechanistic underpinnings that are related to desiccation, it is imperative that more studies incorporate the effects of water loss into disease transmission dynamics.

#### 2.3.4. Reproductive Plasticity is Inherently Related to Maternal Condition and is Likely Influenced by Environmental Conditions

Humidity has been implicated in behavioral alterations that are related to oviposition and breeding site selection [138]. A specific gene, *ppk301*, has been recently implicated in oviposition site selection, where it assists in water- and salt-sensing [139]. Other responses may consist of temperature influences on the oviposition and hatching of *An. quadrimaculatus* eggs [140], as well as influences of temperature and humidity on ovary development [141], oviposition rates, egg production, hatch rate, and adult survival, with subsequent influences on population density [142,143]. Phenotypic plasticity has previously been observed in mosquitoes during gonotrophic dissociation, where blood feeding is undertaken despite cessation in egg production [144]. When considering the propensity for phenotypic plasticity and the environmental influences that are related to mosquito reproduction, water loss from dehydration likely contributes to other reproductive alterations. For example, sub-optimal bloodmeals have been implicated in reductions in vitellogenesis [145] and egg production [86], where similar compromises are found in egg quantity, but not quality, have been observed in dehydrated *C. pipiens* [8]. A potential explanation for uncompromised egg quality is in the equal distribution of calories to mature oocytes regardless of the nutritional stress applied [83]. Therefore, it is intuitive that the size of a bloodmeal determines the number of eggs produced, but it depends upon the nutritional status of the mosquito before the bloodmeal [132]. Unfortunately, causal relationships between dehydration and reproduction are not well documented. Water loss has been repeatedly implicated in blood feeding and mosquito metabolism [6,12,16], which have many known interactions with nutritional reserves and reproduction (Figure 1, Tables S1 and S2). As observed during dry seasons [40], the influences of dehydration on reproduction likely include reduced oviposition, oocyte resorption, gonotrophic cycle alterations, differential utilization of nutritional reserves, as well as bloodmeal size regulation.

#### 2.3.5. Desiccation Tolerance in Mosquito Eggs Fosters Adaptation to Dry Environments

Not only do adult mosquitoes adapt to environmental conditions, cold and desiccation adaptation occurs in *Ae. stegomyia* eggs [146], and would also likely contribute to the evolution of other mosquito species eggs. The selection of eggs with thermal- or desiccation-resistance is likely critical when considering that mosquitoes within the *An. gambiae* complex lay less eggs in the dry season than wet [147], and that traits associated with adaptation to desiccating conditions have been observed in *Ae. notoscriptus*, *Ae. aegypti*, *An. aquasalis*, and *C. quinquefasciatus* eggs [148,149,150,151]. *Aedes* eggs are generally more desiccation resistant than other mosquito genera, which has been credited as an important component in *Aedes* survival and adaptation to dry environments [148]. Specifically, egg desiccation resistance is dependent upon egg melanization and lipids [149,152], serosal cuticle formation [150], size, microclimate where deposited [153], chitin content, egg volume, and eggshell surface density [148]. Some of these factors may be related to genetic background, environmental conditions during development, size of imbibed maternal bloodmeal, and host behavior [148]. Egg desiccation resistance likely contributes to the overall adaptive ability of the species and, consequently, results in an increased risk of dispersion to previously non-endemic areas [151]. In specific, these factors likely promote successful aestivation and overwintering behaviors in mosquitoes, where desiccation-resistant eggs could allow for survival during dry periods.

## 3. Interactions Between Dehydrating Conditions and Vectorial Capacity

### 3.1. Contributions of Behavior to Vectorial Capacity

Recent publications have explicitly noted a lapse in knowledge on several facets of vector ecology, such as the relationships between environmental factors and disease ecology [154], vector-host interactions and virus ecology [155], and a disparity in findings that precipitation positively or negatively correlates to human infection with WNV [34]. It is becoming apparent that the net difference in vectorial capacity must be determined by understanding both the ecological effects and altered biological components of the vector [72]. An improved understanding of how environmental effects, heat, cold, and dehydration directly affect mosquito physiology should be emphasized in future research. Specific attention should be placed on understanding the underlying components that are associated with stress response and compensatory mechanisms that prompt biological responses.

#### 3.1.1. Blood Feeding and Water Content Regulation Influence Disease Transmission Dynamics

Blood feeding dynamics are complex, but, simply put, they are driven by reproduction, hunger, nutritional status, and water content regulation [36]. Rapid dehydration prompts similar expression patterns to that of thermal stress, but slower dehydration results in altered trehalose synthesis in other fly systems [156], which is a major blood sugar in flies [157], and it is known for its desiccation-resistant properties [12,158]. Interestingly, dehydration prompts an increase in trehalose breakdown in *C. pipiens*, which corresponds to a predicted increase in WNV transmission [6]. Likewise, we see a reduction in the trehalose transporter *Ag*TreT1 in *An. gambiae* due to desiccation or heat exposure, which results in a decreased infection of the midgut with oocysts of the parasite *Plasmodium falciparum* [32]. These findings indicate three themes: (1) desiccation similarly affects gene regulation in prominent representative vectors from both the Culicinae and Anophelinae subfamilies, (2) the outcome of realized infection with arthropod-borne disease is partially dependent upon the metrics by which infectivity is determined, and (3) ecological consequences of the underdeveloped and sometimes confounding interactions between dehydration, disease infection, and heat exposure dynamics must be addressed.

#### 3.1.2. Mosquito Humidity Sensing is Influenced by Hydration Status and Likely Contributes to altered Pathogen Transmission

Dry heat has been attributed to the activation of mosquitoes, but moist-heat has been implicated in increased probing behavior [159,160]. As the time devoid of water increases, mosquitoes discriminate less on the source of a bloodmeal [161], and, once a host is found, the feeding rates of mosquitoes in higher temperatures likewise increases [162]. Interestingly, dehydration phenotypes may drive sensing that is associated with humidity and perspiring hosts, both of which have been suggested as important host cues [163,164]. Although humidity sensing in insects, outside of *Drosophila*, is under researched, important candidate genes within the mosquito 2La inversion polymorphism have been identified. These genes include an ionotropic glutamate receptor [118], which is similar to some *D. melanogaster* ionotropic receptors [165] that have been implicated in hygro- or thermo-sensation (such as IR25a, IR40a, or IR93a [166]); and, a heat shock protein (Hsp90) that has also been implicated in increased desiccation tolerance in *C. pipiens* [167]. As many conserved chemosensory genes exist in insects, hygrosensation receptor candidates may also be conserved. For example, an odorant binding protein (Obp59a) contributed to humidity-sensing in *Drosophila* and it was proposed as a candidate for disease control methods as well as a proxy for climate change progress in mosquitoes [168]. Associations between humidity sensing, host-preference, and disease transmission in mosquitoes are severely wanting, but recent studies indicate new focus in the area.

### 3.2. Alternative Perspectives: Utilizing Environmental Stressors as a Proxy for Behavioral and Biological Adaptation to Insecticide Resistance

Stressors, such as dehydration, starvation, temperature, infection status, and insecticide exposure, can contribute to compensatory alterations in factors, such as feeding behavior, survival, vectorial capacity, fecundity, longevity, and activity [6,78,81,82,83,84,169]. Theoretically, these stressors (disregarding insecticide exposure) have acted upon mosquitoes for over 150 million years, in such time, mosquitoes have become exceptionally efficient vectors [116] for a plethora of pathogens. Mosquitoes still possess the ability to establish insecticide resistance and persist although mosquito control methods have existed for less than a millionth of that time. It may be—through inherent susceptibilities, recurrent exposure, and conserved responses to stressors—that mosquitoes have developed an exceptional ability to adapt and overcome stress. These adaptive abilities are of great concern regarding insecticide application, but also provide a unique opportunity to study how mosquitoes respond to a relatively new stressor in reference to preexisting stressors.

Over time, the selective pressures for resistance that are prompted by pesticide administration begin to place the success of control programs at risk, where more than half of the malaria vectors have already developed pyrethroid resistance in less than a single decade [170]. In one such scenario, the LT50 for *An. gambiae* that was treated with deltamethrin increased by 2.6-fold in just over four months [171]. By researching both insecticide and conserved stress responses in mosquitoes, novel strategies regarding stressor combinations and underlying genetic components may be elucidated. Regardless of the stressor, be it insecticidal or environmental, responses should be comprehensively researched and directly compared to one another.

### 3.3. Incorporating the Underlying Mechanisms of Desiccation Dynamics into Disease Transmission Models Would Promote Accuracy and Efficacy

As drought facilitates the amplification and transmission of Saint Louis encephalitis virus and might do the same in other arboviruses, such as Eastern equine encephalitis virus and WNV, further study into these relationships is warranted [52]. Outbreaks may also rise with increasing short-term dehydration periods [172], and by incorporating behavioral phenotypes that are associated with dehydration, a modeled increase in WNV outbreaks was recently proposed [6]. However, caution must be exercised with post-hoc modeling, since fluctuating temperatures are expected to affect mosquitoes markedly differently than constant temperatures [173]; the same is observed with relative humidity [8]. For example, two papers that were authored by the same researcher found that WNV was associated with drought in the spring followed by a wet summer in Florida, U.S.A. [49], but contrarily found that a wet winter and spring followed by a dry summer constituted an increased prevalence of WNV in New York, U.S.A. [51]. In a similar manner, another study correlated the increase in WNV transmission in the eastern and western United States to both above- and below-average rainfall, respectively [48]. An explanation should be warranted when considering the divergence in findings within these studies, but no biological context (e.g., mosquito population differences) or discussion was provided. Without such an explanation or context, valuable data and conclusions may lack power, or confound and misrepresent genuine trends (more on standardization of data is discussed in 4.1.). Appropriately representing data and trends is vitally important when considering the importance of climate change on temperature and humidity alterations.

Global warming has been postulated as a likely driver of malaria distribution in addition to factors, such as human interposition on the environment and increased density in established human civilizations [121,173]. Surprisingly, age and immunity were still lacking from most malaria models in 2013, along with a variety of other factors, including surface water hydrology and host immunity [174]. Other modeling limitations include the absence of age-related interactions [37], assumptions that all members of parasite and vector groups respond similarly to temperature [38], and that dehydration resistance of local populations is not included. Therefore, model inclusion of temperature on survival [175], RH and post-rainfall egg hatch rate on abundance [176], meteorological predictors of temperature and vapor pressure for outbreaks [57], temperature and virus evolution on distribution [25], as well as integrative climatic studies, have been suggested as means to better understand the patterns in natural processes and enhance predictive abilities [177].

The current findings indicate that climatic alterations in rainy and dry seasons have increased the spread of Dengue vectors throughout Indonesia and into higher altitudes that were previously uninhabited, up to 1,200 meters above sea level [123]. Ongoing geographic expansion has also been observed with *Ae. albopictus* in the Pacific region [178], where expansions are especially concerning, given that *Aedes* mosquitoes are remarkably competent vectors for chikungunya virus (CHIKV), DENV, and Zika virus (ZIKV) [178]. Overall, it can be said that seasonal dynamics contribute to WNV prevalence [49,51], and, from a more physiologically-based study, rainy season temperature and relative humidity can contribute to DENV propagation within the mosquito, which contributes to dengue hemorrhagic fever outbreaks [179].

## 4. Caveats and Future Directions in Desiccation-disease Transmission Research

### 4.1. Utilizing Vapor Pressure Deficit and Increasing Data Transparency Would Promote Interstudy Interpretability

Water vapor of the air might be represented by absolute, specific, or relative humidity, as well as by mixing ratio, saturation deficit, dew point, or wet bulb temperature [180] (Table S4). Each of which may result in the conclusion that survival is decreased, constant, or increased by humidity, depending upon which variable is used [37]. A strong interaction has been found to exist between temperature and relative humidity [37,142,181,182], which is especially important when concerning mosquitoes, and other vulnerable arthropods, when considering their susceptibility to water loss [16]. It is known that relative humidity at different temperatures results in markedly different moisture conditions. For example, the average amount of water vapor per volume in “dry” Death Valley, California is comparable to that of the “moist” conditions of Duluth, Minnesota during the same time of year [182]. As evaporation is dependent upon atmospheric moisture conditions, it is most precisely measured by the vapor pressure deficit, which operates independently of temperature, unlike relative humidity [182]. While there are a number of derivations and approximation methods for vapor pressure, approximations for the saturation vapor pressure (P) at a given temperature (T) are straightforward, accurate, and applicable at ecologically relevant temperatures. Buck (1–2) and Tetens (3–4) approximation methods are both recommended for their simplicity, accuracy, and applicability [183,184,185,186,187]:

(1) $$P_{over\;water}=0.61121e^{\left(\left(18.678-\frac{T}{234.5}\right)\left(\frac{T}{257.14+T}\right)\right)}$$

(2) $$P_{over\;ice}=0.61115e^{\left(\left(23.036-\frac{T}{333.7}\right)\left(\frac{T}{279.82+T}\right)\right)}$$

(3) $$P_{over\;water}=0.61078e^{\left(\frac{17.27T}{T+237.3}\right)}$$

(4) $$P_{over\;ice}=0.61078e^{\left(\frac{21.875T}{T+265.5}\right)}$$

where the equations “over water” apply to air temperatures above 0 °C and equations “over ice” apply to air temperatures below 0 °C [184,185,187,188,189]. After calculation of the saturation vapor pressure, vapor pressure of the air ($P_{a}$) can be calculated (5):

(5) $$P_{a}=\frac{RH\times P}{100}$$

and the vapor pressure deficit ($P_{d}$) might be determined by finding the difference between the saturation vapor pressure and the vapor pressure of the air (6) [180,190].

(6) $$P_{d}=P-P_{a}$$

As the vapor pressure deficit increases, the rate at which an organism can lose water to the atmosphere increases, especially in organisms that are highly susceptible to water loss. A substantial loss occurs for mosquitoes, which lose water at all vapor pressures less than saturation [12,191]. For analytical purposes, supplementary worksheets have been attached to assist in the calculation of saturation vapor pressure, vapor pressure of the air, and the resulting vapor pressure deficit from air temperature and relative humidity while using both Buck and Tetens approximations (Tables S5–S7). For interstudy comparisons and meta-analyses, conversions and other equations relating to vapor content of the air have been added (Table S4).

**Figure 2:**
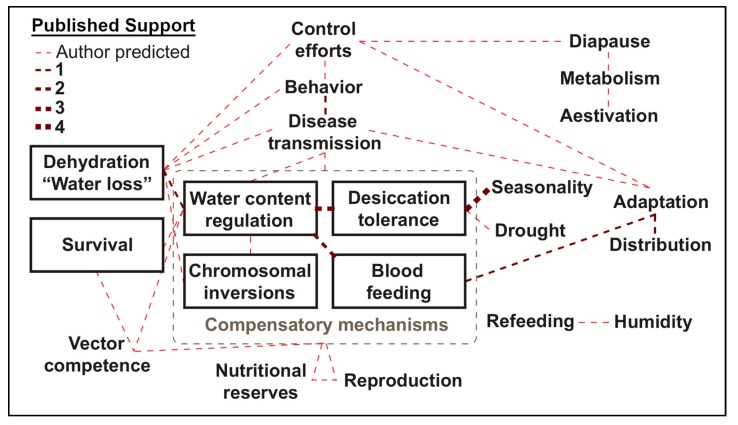
Predicted associations related to the dehydration of mosquitoes as proposed throughout the review. Red dashed lines represent associations solely predicted in this publication (“Author predicted”), blood red dashed lines are author predictions supported by other publications, and the number of supporting publications is represented by line thickness (1–4). Proposed compensatory mechanisms are grouped inside a dashed grey box. All terms and relationships are outlined in Table S3.

Understanding that both temperature and humidity can also independently influence mosquitoes necessitates the appeal for variable independence since desiccation and thermal tolerance in insects share similar mechanisms of resistance, including size, metabolism, and cellular stress alterations [18,156,192]. Especially considering the predicted involvement of dehydration-related factors in numerous facets of mosquito biology, behavior, and disease transmission dynamics (Figure 2, Table S3). Biologically speaking, independence is important because, at extremes, temperature and humidity are both detrimental, but when optimized, either factor enhances most biological processes for mosquitoes [193]. Schmalhausen’s law stresses that, as a population approaches a tolerance limit of one aspect (e.g., thermal), it becomes highly sensitive to minute alterations in other aspects (e.g., desiccation) [194]. Importantly, it is when deviation from the optimal temperature or water vapor pressure occurs that pest control interventions may be used with increased success [195]. However, caution must be exercised, since some assumptions (e.g., assumptions of constant temperature instead of using more ecologically-relevant fluctuating temperatures) may result in improper estimation of mosquito population dynamics [196]. Therefore, it is recommended that temperature and water vapor content of the air be meticulously catalogued, full descriptions of climatic conditions be disclosed [197], and acute attention be paid to microclimatic influence in future publications.

### 4.2. Desiccation Resistance Dynamics are Likely Applicable in Other Arthropod Systems of Interest and May Be Utilized to Foster Comparative Interspecies Analyses

Water loss rates are high in important mosquito disease vectors, such as *C. pipiens, Ae. aegypti, Ae. albopictus, An. arabiensis*, and *An. gambiae* [16]. In general, this water loss often continuously occurs through active mechanisms, such as excretion, respiration, cuticular diffusion [126], defecation, reproduction, secretion, and passively through transpiration [195]. In the black fly, *Simulium arcticum*, water loss through evaporation into the atmosphere was determined by Shipp et al. as a function of differences between the black fly and atmospheric vapor pressures. Evaporation was influenced by factors, including body shape, size, depth of the boundary layer, state of hydration, diffusive path length, and spiracle resistances in the black fly, as well as atmospheric factors, such as hibernacula presence, radiation load, and wind speed [190]. These body and environmental factors, in addition to others, can similarly contribute to mosquito water loss and warrant the utilization of water loss mitigation mechanisms (see Section 2.2 and Section 2.3).

When considering that compensatory behavioral responses in ecological settings, such as the exploitation of microhabitats by mosquitoes, likely allow for decreased desiccation- and temperature-related mortality [37], awareness and inclusion of data from reprieves are also necessary. Consistent with their geographical distribution, *Ae. aegypti* and *Ae. albopictus* possess behavioral mechanisms that are related to feeding, reproduction, and dispersal to likely facilitate survival in unfavorable temperature- and humidity-related field conditions [37]. Similar compensatory behavioral mechanisms are observed in highly dehydration-resistant organisms, such as the American dog tick, *Dermacentor variabilis* [198], and in genetically-similar organisms, such as *Drosophila serrata* and *D. birchii* [199]. Water loss in other arthropods, such as the common and tropical bed bug, *Cimex lectularius* and *C. hemipterus*, is comparably affected by temperature, relative humidity, exposure time, and interactions between temperature and relative humidity [200]. In reality, similar responses to water loss have been observed across a range of arthropod species [198]. Similar responses to high temperatures and vapor pressure deficits between mosquitoes and other arthropods have also been noted. For both western flower thrips, *Frankliniella occidentalis* [197] and several mosquito species [37,91,92,93,143,145], high temperatures and vapor pressure deficits resulted in decreased survival. Therefore, it is probable that these concepts may also be expanded to other dehydration-susceptible organisms, such as, *Ixodes uriae, Ix. scapularius, Ix. ricinus, Xenopsylla confromi, Xe. ramesis, Rhipicephalus annulatus*, and *Ctenocephalides felis* [16], as well as into other fly systems of interest, like *Musca domestica, Drosophila spp., Glossina morsitans*, and *Lucilia cuprina.*

The potential for rehydration via increased blood feeding in mosquitoes might also be comparable to rehydration behaviors that are exhibited by other arthropods. For instance, several spider mite species have been shown to increase consumption of water-rich foods when dehydrated, and the parasitic wasp, *Pachycrepoideus vindemmiae*, increased parasitism of *Drosophila suzukii* pupae to compensate for lost water [201,202]. These dehydration-related behaviors observed in mosquitoes, various spider mites, and *P. vindemmiae* all have reproduction- and survival-associated tradeoffs with water content regulation [6,12,16,201,202]. Interestingly, temperature and starvation also impact spiracular control [203], which underscores the importance of understanding causal relationships and how they are influenced by indirect factors. Environmental adaptations in insects span cold, heat, desiccation, starvation, and numerous other stressors [78]. With many being exposed to the same environmental stressors, some adaptive strategies are shared within insects, as noted above, and they appear to form an integral part of insect biology. Additional exploration of interspecies adaptive similarities would likely offer an increased mechanistic understanding that might be utilized in future control or ecological efforts.

## 5. Conclusions

There is a strong association between humidity and temperature on mosquito mortality, from which desiccation likely contributes to viral transmission in not only arid regions [37], but also temperate or humid climates during dry periods [6]. Desiccation adaptations also dictate the distribution and contribute to disease transmission by *An. gambiae sensu stricto* [85]. Immune evasion by pathogens, such as malaria, is enhanced by co-evolution with the host, but it is paramount that these dynamics be investigated in the appropriate context with the hydration status known in light of the specificity of these interactions [204]. When extrapolating laboratory studies to field research, lab strains must be used with caution [38], and methodological details regarding desiccation (as discussed in 1.2.) must be addressed.

It is advised that vapor pressure deficit, as well as all temperature and humidity data, be recorded frequently with ample consideration for the existence of microhabitat influence to promote productivity in the field and clarity in results. It is important to reiterate that desiccation affects genetic, biological, and behavioral components within prominent vectors from both the Culicinae and Anophelinae subfamilies. Studies including behavior, field observation, and underlying biology of behavioral plasticity are all wanting but necessary for improving current and proposing new intervention techniques [205]. Overall, understanding the compensatory mechanisms by which mosquitoes curtail dehydration stress will offer an increased understanding regarding important components of mosquito biology, such as survival, reproduction, and vectorial capacity. Continuance with this research might be utilized to better understand, and potentially decrease, the impact of disease in the world’s deadliest animals.

## Supplementary Materials

The following are available online at https://www.mdpi.com/2075-4450/10/11/375/s1, Table S1: Relationships between terms associated with three or more publications, Table S2: Term associations with two or less publications (including references [206,207,208,209,210,211,212,213]), Table S3: Factor relationships predicted within this review, Table S4: Expressions of water vapor as adapted from Slatyer [180], Table S5: Interactive table for calculation of Buck and Tetens approximations for saturation vapor pressure, vapor pressure of the air, and vapor pressure deficit from a known temperature (either above or below 0 °C) and relative humidity, Table S6: Interactive table for calculation of water vapor approximations from relative humidity and temperatures (above 0 °C) for multiple data entries, Table S7: Interactive table for calculation of water vapor approximations from relative humidity and temperatures (below 0 °C) for multiple data entries.
