# Supplementary material for: Biological Adaptations Associated with Dehydration in Mosquitoes
Source: Insects. 2019 Oct 28;10(11):375. doi: 10.3390/insects10110375 (PMC6920799; doi:10.3390/insects10110375)
Supplement: Supplementary file 1 [file insects-10-00375-s001.zip › Supplementary_Tables 1.docx]

**Table 1.** Relationships between terms associated with three or more publications. Primary and secondary terms refer to the factors involved, effect and certainty refer to the relationship and strength between the two factors, and references refer to the publication number as cited throughout the review.

| **Primary** | **Effect** | **Certainty** | **Secondary** | **Additional Info** | **Reference(s)** |  |
| --- | --- | --- | --- | --- | --- | --- |
| Humidity | influences | known | Survival | e.g. eggs / relative humidity | 6,37,91,92,93,125,126,143,146,199 | (10) |
| Seasonality | influences | known | Adaptation |  | 37,40,63,87,107,117,118,122,124 | (9) |
| Temperature | influences | known | Survival |  | 37,91,92,93,143,146,199 | (7) |
| Chromosomal inversions | influence | known | Desiccation tolerance |  | 9,117,118,119,120,121 | (6) |
| Humidity | influences | known | Temperature |  | 37,143,160,161,182,195 | (6) |
| Temperature | influences | known | Humidity |  | 37,143,160,161,182,195 | (6) |
| Chromosomal inversions | influence | known | Insecticide resistance |  | 208,209,210,211 | (4) |
| Desiccation tolerance | impacts | known | Distribution | macro- and micro- | 9,106,148,205 | (4) |
| Genes | influence | known | Desiccation tolerance | eggs: hsp70/hsp90 RNAi | 32,104,149,167 | (4) |
| Humidity | alters | known | Activity | relative humidity | 6,88,160,161 | (4) |
| Seasonality | influences | known | Desiccation tolerance | i.e. eggs | 148,149,150,153 | (4) |
| Seasonality | influences | known | Disease transmission |  | 56,58,123,172 | (4) |
| Malpighian Tubules | regulate | known | Water loss | via diuresis | 10,19,23 | (3) |
| Behavior | increases | predicted | Disease reservoir |  | 14,100,207 | (3) |
| Behavior | influences | known | Blood feeding |  | 37,83,121 | (3) |
| Blood feeding | influences | known | Water content regulation |  | 6,19,20 | (3) |
| Blood feeding | influences | known | Water loss | e.g. influx/excretion | 6,19,20 | (3) |
| Blood feeding | prompts | known | Egg production |  | 8,86,138 | (3) |
| Chromosomal inversions | regulate | known | Genes |  | 18,118,121 | (3) |
| Chromosomal inversions | influence | predicted | Adaptation |  | 18,118,122 | (3) |
| Chromosomal inversions | facilitate | predicted | Distribution |  | 18,118,122 | (3) |
| Dehydration | prompts | known | Metabolism | i.e. trehalose | 6,32,157 | (3) |
| Dehydration | influences | predicted | Disease transmission |  | 6,37,49 | (3) |
| Desiccation tolerance | reduces | known | Water loss |  | 11,12,13 | (3) |
| Desiccation tolerance | increases | known | Survival |  | 12,37,103 | (3) |
| Diapause | increases | known | Desiccation tolerance |  | 11,12,13 | (3) |
| Disease | causes | known | Health burden |  | 2,4,5 | (3) |
| Disease | causes | known | Socioeconomic burden |  | 2,4,5 | (3) |
| Drought | influences | known | Development | larvae 🡪 adult; less flushing | 54,59,96 | (3) |
| Seasonality | influences | known | Metabolism |  | 106,124,129 | (3) |
| Speciation | drives | known | Desiccation tolerance |  | 16,103,124 | (3) |
| Temperature | influences | known | Hatching |  | 142,143,144 | (3) |
| Temperature | influences | known | Oviposition |  | 142,143,144 | (3) |
| Water content regulation | influences | known | Desiccation tolerance |  | 91,126,133 | (3) |
