# Supplementary material for: Biological Adaptations Associated with Dehydration in Mosquitoes
Source: Insects. 2019 Oct 28;10(11):375. doi: 10.3390/insects10110375 (PMC6920799; doi:10.3390/insects10110375)
Supplement: Supplementary file 1 [file insects-10-00375-s001.zip › Supplementary_Tables 3.docx]

**Table 3.** Factor relationships predicted within this review. Primary and secondary terms refer to the factors involved, effect refers to the influence of the primary factor on the secondary factor, and reference support refers to the number of external publications referencing the association.

| **Primary** | **Effect** | **Secondary** | **Reference Support** |
| --- | --- | --- | --- |
| Seasonality | influences | Desiccation tolerance | 4 |
| Water content regulation | influences | Desiccation tolerance | 3 |
| Water content regulation | influences | Blood feeding | 2 |
| Adaptation | influences | Blood feeding | 1 |
| Adaptation | influences | Distribution | 1 |
| Behavior | influences | Disease transmission | 1 (predicted) |
| Water content regulation | influences | Survival | 1 (predicted) |
| Adaptation | influences | Disease transmission | 0 |
| Behavior | alters | Water loss | 0 |
| Compensatory mechanisms | influence | Disease transmission | 0 |
| Compensatory mechanisms | influence | Nutritional reserves | 0 |
| Compensatory mechanisms | influence | Reproductive capacity | 0 |
| Compensatory mechanisms | influence | Vector competence | 0 |
| Dehydration | influences | Control efforts | 0 |
| Drought | influences | Desiccation tolerance | 0 |
| Humidity | influences | Refeeding | 0 |
| Survival | increases | Vector competence | 0 |
| Water content regulation | influences | Disease transmission | 0 |
| Water content regulation | influences | Vector competence | 0 |
| Water content regulation | influences | Water loss | 0 |
| Adaptation | complicates | Control efforts | 0 |
| Aestivation | influences | Metabolism | 0 |
| Diapause | influences | Metabolism | 0 |
| Diapause | influences | Control efforts | 0 |
| Chromosomal inversions | influence | Water content regulation | 0 |
| Chromosomal inversions | influence | Dehydration | 0 |
| Nutritional reserves | influence | Reproduction | 0 |
| Dehydration | influences | Disease transmission | 0 |
| Behavior | influences | Control efforts | 0 |
