# Supplementary material for: Biological Adaptations Associated with Dehydration in Mosquitoes
Source: Insects. 2019 Oct 28;10(11):375. doi: 10.3390/insects10110375 (PMC6920799; doi:10.3390/insects10110375)
Supplement: Supplementary file 1 [file insects-10-00375-s001.zip › Supplementary_Table_Descriptions.docx]

**Table S1:** Relationships between terms associated with three or more publications. Primary and secondary terms refer to the factors involved, effect and certainty refer to the relationship and strength between the two factors, and references refer to the publication number as cited throughout the review.

**Table S2:** Term associations with two or less publications. Primary and secondary terms refer to the factors involved, effect and certainty refer to the relationship and strength between the two factors, and references refer to the publication number as cited throughout the review.

**Table S3:** Factor relationships predicted within this review. Primary and secondary terms refer to the factors involved, effect refers to the influence of the primary factor on the secondary factor, and reference support refers to the number of external publications referencing the association.

**Table S4:** Expressions of water vapor as adapted from Slatyer [180]. Equations for the vapor content of the air are included to mathematically represent the similarities and differences between commonly used expressions in the literature.

**Table S5:** Interactive table for calculation of Buck and Tetens approximations for saturation vapor pressure, vapor pressure of the air, and vapor pressure deficit from a known temperature (either above or below 0°C) and relative humidity. Entering values for air temperature and relative humidity (C2-C3 for temperatures above 0°C; D2-D3 below 0°C) will calculate all vapor pressure variables for both Buck (> 0°C, C4-C6; < 0°C, D4-D6) and Tetens (> 0°C, C9-C11; < 0°C, D9-D11).

**Table S6:** Interactive table for calculation of water vapor approximations from relative humidity and temperatures (above 0°C) for multiple data entries. Calculates Buck approximations for vapor pressure of the air (B_air_), saturation vapor pressure (B_saturation_), vapor pressure deficit (B_deficit_), and Tetens approximations for vapor pressure of the air (T_air_), saturation vapor pressure (T_saturation_), vapor pressure deficit (T_deficit_).

**Table S7:** Interactive table for calculation of water vapor approximations from relative humidity and temperatures (below 0°C) for multiple data entries. Calculates Buck approximations for vapor pressure of the air (B_air_), saturation vapor pressure (B_saturation_), vapor pressure deficit (B_deficit_), and Tetens approximations for vapor pressure of the air (T_air_), saturation vapor pressure (T_saturation_), vapor pressure deficit (T_deficit_).
